# GROUND REACTION FORCE IN SIT-TO-STAND MOVEMENT PREDICT SARCOPENIA

**DOI:** 10.1093/geroni/igae098.3992

**Published:** 2024-12-31

**Authors:** Kazuki Ota, Jieun Yoon, Kyohei Shibuya, Mariko Miyashita, Tomoko Miyama, Tomohiro Okura

**Affiliations:** University of Tsukuba, Tsukuba, Ibaraki, Japan; University of Tsukuba, Tsukuba, Ibaraki, Japan; University of Tsukuba, Tsukuba, Ibaraki, Japan; Tanita Corporation, Itabashi, Tokyo, Japan; Tanita Corporation, Itabashi, Tokyo, Japan; University of Tsukuba, Tsukuba, Ibaraki, Japan

## Abstract

New method for evaluating lower-limb muscle strength has been attempted using ground reaction force during sit-to-stand movement. The specific variables used are the vertical peak force, the rate of force development (RFD), and the stable time. These variables have also been shown to be associated with physical function and experience of falling. However, their relationship to sarcopenia, the loss of skeletal muscle mass and muscle strength, and predictors of sarcopenia have not been investigated. In this study, we aimed to investigate the relationship between ground reaction force during sit-to-stand movement and sarcopenia diagnostic factors, as well as identify predictors of sarcopenia. The subjects were 1462 people aged 46-89 years (71.0±8.9 years). The sarcopenia was determined by grip strength, waking speed, and skeletal muscle mass (SMI) index. Force variables were calculated using the Zaritz (BM-220, Tanita Corporation). The statistical methods used was the Pearson correlation coefficient test, with P < 0.05. In addition, ROC curve analysis was performed, the area under the curve (AUC) and accuracy were calculated. The results showed that there were 1411 people without sarcopenia and 51 people with sarcopenia. Significant positive correlations were found between grip strength, walking speed, and SMI index and vertical peak force (r = 0.446, 0.306, 0.338), RFD (r = 0.264, 0.389, 0.159). Furthermore, the RFD had high discriminatory accuracy (AUC: 0.841, accuracy: 83.3 %). These results suggest that ground reaction force in sit-to-stand movement is a useful predictor of sarcopenia.

